# Computational modelling of reinforcement learning and functional neuroimaging of probabilistic reversal for dissociating compulsive behaviours in gambling and cocaine use disorders

**DOI:** 10.1192/bjo.2023.611

**Published:** 2023-12-11

**Authors:** Katharina Zühlsdorff, Juan Verdejo-Román, Luke Clark, Natalia Albein-Urios, Carles Soriano-Mas, Rudolf N. Cardinal, Trevor W. Robbins, Jeffrey W. Dalley, Antonio Verdejo-García, Jonathan W. Kanen

**Affiliations:** Department of Psychology, University of Cambridge, UK; Behavioural and Clinical Neuroscience Institute, University of Cambridge, UK; and the Alan Turing Institute, London, UK; Department of Personality, Assessment and Psychological Treatment, Universidad de Granada, Spain; and Mind, Brain and Behavior Research Center, Universidad de Granada, Spain; Department of Psychology and Djavad Mowafaghian Centre for Brain Health, University of British Columbia, Canada; Cognitive Neuroscience Unit, School of Psychology, Deakin University, Australia; Department of Psychiatry, Bellvitge Biomedical Research Institute-IDIBELL, Spain; Department of Social Psychology and Quantitative Psychology, University of Barcelona, Spain; and CIBERSAM, Carlos III Health Institute, Madrid, Spain; Behavioural and Clinical Neuroscience Institute, University of Cambridge, UK; Department of Psychiatry, University of Cambridge, UK; and Liaison Psychology, Cambridgeshire and Peterborough NHS Foundation Trust, UK; Department of Psychology, University of Cambridge, UK; and Behavioural and Clinical Neuroscience Institute, University of Cambridge, UK; Department of Psychology, University of Cambridge, UK; Behavioural and Clinical Neuroscience Institute, University of Cambridge, UK; and Department of Psychiatry, University of Cambridge, UK; School of Psychological Sciences, Monash University, Australia; and Turner Institute for Brain and Mental Health, Monash University, Australia

**Keywords:** Cocaine use disorder, gambling disorder, reinforcement learning, prediction error, expected value

## Abstract

**Background:**

Individuals with cocaine use disorder or gambling disorder demonstrate impairments in cognitive flexibility: the ability to adapt to changes in the environment. Flexibility is commonly assessed in a laboratory setting using probabilistic reversal learning, which involves reinforcement learning, the process by which feedback from the environment is used to adjust behavior.

**Aims:**

It is poorly understood whether impairments in flexibility differ between individuals with cocaine use and gambling disorders, and how this is instantiated by the brain. We applied computational modelling methods to gain a deeper mechanistic explanation of the latent processes underlying cognitive flexibility across two disorders of compulsivity.

**Method:**

We present a re-analysis of probabilistic reversal data from individuals with either gambling disorder (*n* = 18) or cocaine use disorder (*n* = 20) and control participants (*n* = 18), using a hierarchical Bayesian approach. Furthermore, we relate behavioural findings to their underlying neural substrates through an analysis of task-based functional magnetic resonanceimaging (fMRI) data.

**Results:**

We observed lower ‘stimulus stickiness’ in gambling disorder, and report differences in tracking expected values in individuals with gambling disorder compared to controls, with greater activity during reward expected value tracking in the cingulate gyrus and amygdala. In cocaine use disorder, we observed lower responses to positive punishment prediction errors and greater activity following negative punishment prediction errors in the superior frontal gyrus compared to controls.

**Conclusions:**

Using a computational approach, we show that individuals with gambling disorder and cocaine use disorder differed in their perseverative tendencies and in how they tracked value neurally, which has implications for psychiatric classification.

## Background

The diagnostic criteria for both substance use disorder (SUD) and gambling disorder in the DSM-5 include unsuccessful attempts to stop substance misuse or gambling, jeopardising relationships and educational/career opportunities, and financial troubles arising as a consequence of the disorder.^1^ Compulsivity, a key feature of both gambling disorder and SUD, is defined as persistent actions inappropriate for a given situation, which have no clear relationship to the overall goal and frequently result in undesirable consequences.^2^ Gambling disorder and SUD are disorders of compulsivity and their behavioural phenotypes may overlap, but also diverge in certain aspects.^3^ Gaining a clearer definition of these phenotypes could inform the development of new treatments for disorders of compulsivity.

A further common feature of gambling disorder and SUD is behavioural inflexibility, defined as a deficit in adjusting behaviour based on changes in environmental feedback.^4,5^ Individuals with a stimulant-related SUD exhibit higher rates of perseverative responding following a contingency change during probabilistic reversal learning (PRL), a paradigm used to investigate cognitive flexibility.^6^ During this task, individuals learn which action is associated with reward through trial and error. Following changes in stimulus contingencies, individuals need to flexibly adjust behaviour. Indeed, reversal learning is impaired in rats and monkeys following prolonged exposure to cocaine.^7,8^

Patients with gambling disorder, in comparison, show difficulties in learning novel stimulus-outcome associations following contingency changes during reversal learning.^4^ Following repeated negative feedback, patients with gambling disorder tend to stay rather than switch their response, or switch prematurely after little or no negative feedback during PRL.^5^ Individuals with gambling disorder perform significantly worse than healthy controls on the Intra-/Extra-Dimensional Set Shifting test, which assays higher-order cognitive flexibility, with impairments observed at the extra-dimensional shift stage (requiring the most flexibility).^9^ In a meta-analysis of participants diagnosed with gambling disorder on the related Wisconsin Card Sorting Test, patients made more perseverative errors than healthy controls.^10^ Overall, it is evident that individuals with gambling disorder are impaired on cognitive flexibility tasks and have greater perseverative tendencies, similar to individuals with SUD.

## Reinforcement learning modelling

Reinforcement learning is the process by which positive and negative feedback from the environment is used to adjust behaviour, to maximise rewards and minimise punishment.^11^ In recent years, reinforcement learning models have been used increasingly to gain deeper insights into the latent mechanisms underlying PRL on a trial-by-trial basis, which are represented by model parameters. One example of such a parameter is the exploration versus exploitation parameter, which reflects the extent to which learned values contribute to choice behaviour. ‘Stickiness’ parameters track the tendency to repeatedly choose the same stimulus regardless of outcome (i.e. ‘stimulus stickiness’) or the tendency to repeat choices in the same location as before, irrespective of outcome (i.e. ‘side stickiness’). These stickiness parameters fractionate the construct of perseveration as they parse different types of repetitive behaviours; for example, toward a location or a stimulus. Additionally, standard measures of perseveration assess behaviour following a contingency reversal, whereas stickiness accounts for a tendency to repeat behaviours across all trials. Reward and punishment learning rates can also be determined via reinforcement learning models, which index the speed at which the expected value of a choice is updated after a better or worse than expected outcome (reward or punishment prediction error). Indeed, reinforcement learning impairments following drug use and withdrawal have been demonstrated in rodents and humans. In rats, increased exploitation and stickiness have been reported after cocaine self-administration.^12^ Humans with SUD have also been found to have higher levels of stickiness, alongside greater punishment learning rates and lower reward learning rates.^13^ Critically, the reinforcement learning fingerprint during PRL in gambling disorder has not been elucidated.

## Neural substrates of reward processing in disorders of compulsivity

Cocaine use disorder (CUD) has been associated with altered reward processing linked to differences in frontostriatal activity. For example, a study employing functional magnetic resonance imaging (fMRI) has found that individuals diagnosed with CUD exhibited lower blood–oxygen level dependent (BOLD) signals in the orbitofrontal cortex (OFC) than control participants, following monetary gains on a forced-choice task containing three monetary value conditions.^14^ Neural activity is also known to be altered in patients with SUD during PRL, such as in the middle frontal gyrus (MFG) and caudate nucleus, areas known to contribute to performance on this task.^6,15^ A meta-analysis of 52 studies reported that the OFC is hypoactive following detoxification in participants with CUD across different decision-making tasks.^16^ Thus, it is evident that activity of striatal and prefrontal cortical (PFC) regions is altered in CUD.

fMRI studies in individuals with gambling disorder have also found differential recruitment of PFC areas during reward-based tasks.^3^ The ventromedial PFC (vmPFC), an area activated during monetary reward tasks in healthy individuals that is important for reward processing, shows lower task-related activation in gambling disorder.^17^ On the Iowa Gambling Task, greater activity in individuals with gambling disorder during high-risk choices has been reported in the right caudate, OFC, vmPFC, superior frontal gyrus (SFG), amygdala and hippocampus.^18^ Furthermore, lower activity in the right ventrolateral PFC (vlPFC) has been linked to higher levels of perseveration on a PRL task.^19^ These findings point to altered reward processing in gambling disorder and suggest the involvement of cortical areas such as the vmPFC and OFC, as well as subcortical structures; several areas overlap with those also affected in CUD. However, the neural substrates underlying reinforcement learning in gambling disorder and CUD are not clearly defined. In rats, stickiness positively correlated with resting-state fMRI activity between the medial OFC (mOFC), PFC and subcortical structures.^20,21^ In humans, the link between reinforcement learning behaviour and neural activity in these clinical populations has not yet been established.

## Study summary

Here, we present a re-analysis of a previously published data-set,^22^ using novel computational methods. Individuals with CUD, gambling disorder and controls completed a PRL task in an fMRI scanner. In the previous publication arising from this data-set, conventional PRL measures were calculated and compared between the groups. There, it was reported that a behavioural variable reflecting the perseveration error rate was increased in the CUD group, with no differences observed in the gambling disorder group. Additionally, both patient groups had lower vlPFC activation when shifting responding following a reversal. In the new analysis presented here, reinforcement learning models are employed to reveal latent processes underlying behaviour on the PRL task, via a potentially more sensitive trial-by-trial approach. Through the fMRI data, the reinforcement learning parameters can be linked to their associated neural substrates. To our knowledge, no previous studies have analysed PRL data from patients with gambling disorder, using reinforcement learning models. Based on our recent work that showed the concept of stickiness was critical for dissociating other disorders of compulsivity,^13^ we hypothesised that individuals with gambling disorder and CUD would show impairments in stickiness, and that stickiness would be greater in CUD. Neurally, we predicted that activity in the OFC would be linked to the reward learning rate, and that medial PFC and dorsal striatal activity would reflect the stickiness parameter.

## Method

### Participants

Fifty-six participants took part in this study. These comprised 18 healthy controls who did not meet any of the criteria for an Axis 1 or 2 disorder; 18 individuals who met the DSM-IV-TR criteria for pathological gambling and 20 individuals who met the criteria for cocaine dependence. Here, we use the terms CUD and gambling disorder, which are the current nomenclature in the DSM-5, rather than cocaine dependence and pathological gambling, as used in the DSM-IV-TR.^1^

Basic behavioural data in association with fMRI findings from this study have previously been published.^22^ The authors assert that all procedures contributing to this work comply with the ethical standards of the relevant national and institutional committees on human experimentation and with the Helsinki Declaration of 1975, as revised in 2008. All procedures involving human patients were approved by the Ethics Committee for Research in Humans, University of Granada, Spain (approval number CEIH 2009/052). Participants signed an informed consent form to confirm their voluntary participation and were all equally reimbursed for their participation. Written informed consent was obtained from all participants. Please see Supplementary Material available at https://doi.org/10.1192/bjo.2023.611 for further information on participant recruitment.

### PRL task

This task was similar to the PRL task used by Cools et al.^15^ Two abstract, coloured stimuli were presented on the right and left side of the visual display. Stimulus location was randomised. At the beginning of the tasks, everyone was informed that one stimulus was the ‘correct’ stimulus (CS+), and the other stimulus was the ‘incorrect’ stimulus (CS−). Participants had to learn the correct and incorrect stimulus through a trial-and-error approach. The CS+ resulted in a reward on only 85% of the trials, whereas the CS− was rewarded 15% of the time. Following 10–15 correct responses, the contingencies were reversed. All participants were trained on the PRL task outside the scanner before the initial scan, for which different stimuli were used. During scanning, there were three consecutive blocks that consisted of ten discriminations (nine reversals), with a duration of 11 min per block.

Magnetic-resonance-compatible liquid-crystal display goggles were used to present the stimuli (Resonance Technology, California, USA). All responses were recorded with the Evoke Response Pad System (Resonance Technology). This button box was located on the participant's chest. The duration of stimulus presentation was 2000 ms. If participants failed to respond during this time, a ‘too late’ message was presented. Following a ‘correct’ response, a green smiley face was presented, and following an ‘incorrect’ response, a red sad face was shown. Feedback was presented for 500 ms, during which time the stimulus remained on the screen. Following feedback presentation, there was a variable inter-trial interval, which was adjusted by the program, for a final interstimulus interval duration between stimuli of 3253 ms. This interstimulus interval duration was selected to enable a precise desynchronisation from the repetition time (2000 ms).

### Reinforcement learning modelling

The PRL data was modelled with reinforcement learning models, using a hierarchical Bayesian approach. Six different models were run to test different combinations of model parameters, implemented through Stan for R (RStan version 2.26.1; see https://mc-stan.org/users/interfaces/rstan).^23^

Q values were updated on a trial-by-trial basis, according to the following equation:1
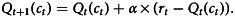


*Q*_*t*+1_(*c_t_*) is the expected value for the next trial based on the stimulus that is chosen on the current trial, *Q_t_*(*c_t_*) is the expected value of the choice taken on the current trial, *α* is the learning rate and *r_t_* is the reinforcement on trial *t* (1 for reward and 0 for punishment). The learning rate influences how much the participant updates the *Q* value based on the prediction error *r_t_* − *Q_t_*(*c_t_*), with higher *α* driving faster learning.

The probability of making one of two choices, given the *Q*-values for each, was calculated using the softmax decision rule:2
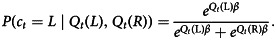


*Q_t_*(*L*) and *Q_t_*(*R*) are the *Q*-values of the left and right stimuli, and *β* is the reinforcement sensitivity parameter, which determines to what extent the participant is driven by its reinforcement history (versus random choice). Six models were tested and the parameters from the winning model were subsequently used for data simulation. Further information on these methods can be found in the Supplementary Material.

### First-level models

Information on image acquisition and pre-processing can be found in the Supplementary Material. First-level linear models were fit through FEAT (FSL for Linux, Analysis Group, FMRIB, Oxford, UK; see https://fsl.fmrib.ox.ac.uk/fsl/fslwiki/FslInstallation/Linux).^24^ A first-level model was fit for each run, and included the following event types: (a) reward expected value, (b) positive reward prediction error (RPE), (c) negative RPE, (d) punishment expected value, (e) positive punishment prediction error (PPE), (f) negative PPE and (g) response/feedback presentation. The RPE is when the prediction error is greater than 0 (when the outcome is better than the expected value) and is positive when there is a reward, and negative if the reward is omitted. The PPE is the prediction error is below 0 (outcome is worse than the expected value). Similar to the RPE, it is positive if there is a reward, and negative when these is no reward. RPEs take values between 0 and 1, whereas PPEs are between 0 and −1. Expected values and prediction errors were extracted for each trial from the winning *Q*-learning model. Explanatory variables 1–6 were based on the extracted values of prediction error and expected *Q*-values as calculated in Eq. 1. The model was based on an analysis presented previously.^25^ Event types 1 and 4 were fitted during stimulus presentation, whereas 2, 3, 4 and 6 were fitted during feedback presentation. These first-level model regressors represent trial-level measures, whereas the reinforcement learning parameters introduced in the previous section are participant-level measures. Expected values, RPEs and PPEs were added as parametric modulators for the respective event types. Six movement parameters (*x*, *y*, *z*, pitch, roll, yaw) were incorporated into the model, which resulted from the image realignment to control for movement artefacts.

### Higher-level models

The first-level models were averaged across the three runs for each participant, resulting in the second-level models. Third-level, mixed-effects, whole-brain analyses involving one-factor, three-level analyses of variance with *post hoc t*-tests and cluster thresholding with a Z threshold of ±3.1 and *P* < 0.05 were used to investigate the contrasts for each event type.^26^ Subsequently, an analysis of covariance (ANCOVA) was run as an additional exploratory analysis. In the ANCOVA, model parameters from the best-fitting reinforcement learning model were extracted for each participant and included as predictors. The aim of this analysis was to investigate group differences in the correlation between activity in a given region and a reinforcement learning parameter (i.e. a group × reinforcement learning parameter interaction). Reinforcement learning parameters were also correlated with the BOLD signal from all participants, regardless of group. FSLeyes (FSL) was used to generate figures.^27^ In all figures, the right and left sides are inverted from the observer's perspective (according to standard radiological convention).

## Results

### Demographic information

There were no significant differences in age, gender, IQ, handedness or years of education between the groups (Table 1).^22^

**Table 1:** Demographic information

	Healthy controls (*n* = 18)	Gambling disorder (*n* = 18)	Cocaine use disorder (*n* = 20)	Group comparisons
Mean age, years (s.d.)	31.2 (4.7)	33.6 (8.0)	34.3 (6.9)	*F*(2,54) = 1.43, *P* = 0.35
Gender (female)	1	2	1	*χ*^2^(2,56) = 0.59, *P* = 0.75
Verbal IQ, mean (s.d.)	106.9 (9.0)	102.7 (7.4)	100.9 (7.6)	*F*(2,54) = 2.31, *P* = 0.082
Years of education, mean (s.d.)	10.6 (1.9)	10.3 (2.1)	9.8 (1.7)	*F*(2,54) = 1.37, *P* = 0.47
Handedness (left)	1	1	4	*χ*^2^(2,56) = 2.80, *P* = 0.25

### Selecting the winning model

Table 2 reports the results from the six reinforcement learning models tested and model comparison measures. Satisfactory model convergence was confirmed, as all parameters and contrasts had a potential scale reduction factor of <1.1, with the maximum value being 1.006.

**Table 2:** Model comparison summary

Model	Rank	Parameters	Log marginal likelihood	Log posterior *P*
1	4	*α*, *β*	−13 159.06	−573.18
2	5	*α, β, κ*_stim_	−13 018.42	−432.54
3	3	*α*_rew_, *α*_non-rew_, *β*	−13 130.08	−544.21
4	2	*α*_rew_, *α*_non-rew_, *β*, *κ*_stim_	−13 003.72	−417.85
5	6	*α*_rew_, *α*_non-rew_, *β*, *κ*_side_	−13 168.48	−582.60
6	1	*α*_rew_, *α*_non-rew_, *β*, *κ*_side_, *κ*_stim_	−11 139.35	0.000

Models were assumed to be equiprobable *a priori*. *α*, learning rate; ^*α*^rew, learning rate from rewarded trials; ^*α*^non-rew, learning rate from non-rewarded trials; β, reinforcement sensitivity; ^*κ*^side, side stickiness; ^*κ*^stim, stimulus stickiness.

The winning model (model 6) contained five parameters: the reward learning rate *α*_rew_, representative of how quickly an individual updates (increases) *Q*-values in response to positive feedback; the punishment learning rate *α*_pun_, reflecting how quickly an individual updates (decreases) the *Q*-value following punishment; reinforcement sensitivity *β*, also known as the exploitation versus exploration or inverse temperature parameter; stimulus stickiness *κ*_stim_, which is the tendency to select the same stimulus regardless of outcome, and side stickiness *κ*_side_, which is the tendency to select the same side regardless of outcome.

### Reinforcement learning results

**Fig. 1:**
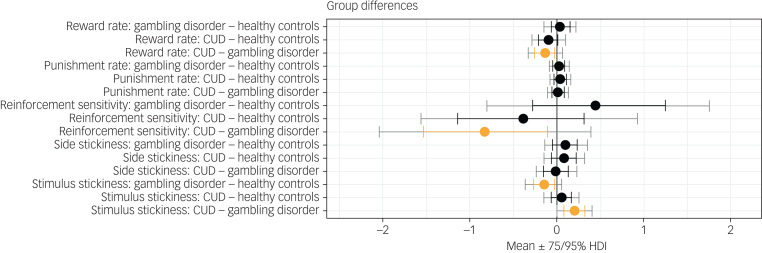
Results from the hierarchical Bayesian winning reinforcement learning model, showing differences in group mean parameters. Orange indicates 0 ∉ 75% HDI. CUD, cocaine use disorder; HDI, highest density interval.

Figure 1 shows results of the hierarchical Bayesian reinforcement learning analysis. Neither the reward learning rate nor the punishment learning rate were affected in gambling disorder or CUD when compared with healthy controls. However, there was evidence that the reward learning rate *α*_rew_ was lower in the CUD group than the gambling disorder group (difference in parameter per-group mean, posterior 75% highest density interval (HDI) excluding zero). Reinforcement sensitivity was lower in the CUD group compared with the gambling disorder group, reflecting more exploratory behaviour in the CUD group, as well as higher *κ*_stim_ in the CUD group compared with the gambling disorder group (group differences, 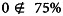 HDI). Side stickiness was not different in either patient group compared with the control group (no group differences, 0 ∈  75% HDI). There was evidence for lower stimulus stickiness at 75% HDI in the gambling disorder group compared with healthy controls (group difference, 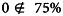 HDI). There were no differences in the CUD group when compared with the control group (no group differences, 0 ∈  75% HDI). To summarise, we found evidence for the stimulus stickiness parameter *κ*_stim_ being lower in the gambling disorder group compared with healthy controls. No differences at 95% HDI were observed. We note that 95% HDI provides stronger evidence for there being group differences than 75% HDI; however, 75% HDI is also considered to provide sufficient evidence and has been used as a threshold in previous studies.^28^

### Simulations

The parameters from the winning reinforcement learning model were used to simulate the behavioural data and determine whether this model could replicate the behaviour observed initially via raw data measures. When these data were analysed with a conventional approach to extract raw data measures such as the proportion of correct responses trials to criterion and number of perseverative responses, no statistically significant differences between the groups were found. These findings thus align with the results for the conventional behavioural measures presented in Verdejo-Garcia et al,^22^ suggesting that the model was able to reproduce the behavioural dynamics on this task. The results can also be seen in the Supplementary Material.

### Brain activity during reward and punishment expected value tracking in gambling disorder

**Fig. 2:**
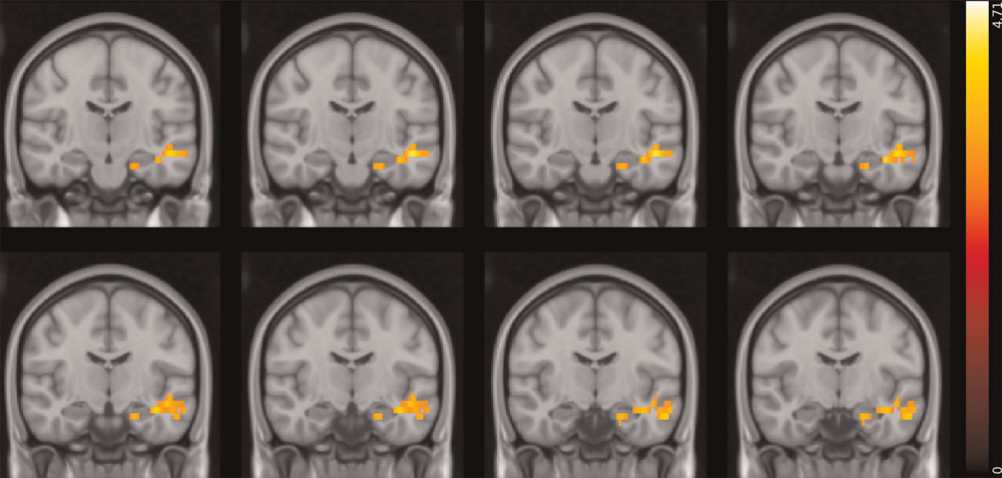
Reward expected value tracking: differences between healthy controls and participants with gambling disorder (Montreal Neurological Institute coordinates: *Y* = −18 to −11). Activity was higher in the gambling disorder group in the areas indicated. Colour bar on the right-hand side represents the *t*-statistic.

The model fitted to the task-based fMRI data included seven explanatory variables, as above: (a) reward expected value, (b) positive RPE, (c) negative RPE, (d) punishment expected value, (e) positive PPE, (f) negative PPE and (g) response/feedback presentation. We found differences in the neural responses to reward and punishment expected value in the gambling disorder group compared with controls. Specifically, we observed that when tracking reward expected value, individuals with gambling disorder had greater activations in the amygdala; hippocampus; parahippocampal gyrus; lateral occipital cortex; superior, inferior and middle temporal gyri; posterior cingulate gyrus and precuneus than healthy controls (Fig. 2 and Table 3). These effects were only observed in the left hemisphere.

**Table 3:** Summary of peak functional magnetic resonance imaging activity for the reward expected value controls versus gambling disorder contrast

Name	Brodmann area	Side	MNI coordinates (*X, Y, Z*)	Number of voxels	Volume (mm^3^)	Mean *Z-*statistic
Middle temporal gyrus	21	Left	−57, −6, −17	365	5893	3.63
Precuneus	7	Left	−3, −66, 33	224	3617	3.68
Cingulate gyrus	24, 32	Left	−9, −50, 27	182	2939	3.71
Superior temporal gyrus	22, 42	Left	−46, −14, −8	112	1808	3.49
Lateral occipital cortex	19	Left	−57, −62, −6	97	1566	3.79
Hippocampus	28	Left	−21, −10, −24	81	1308	3.45
Amygdala	–	Left	−23, −5, −17	68	1098	3.41
Parahippocampal gyrus	27	Left	−17, −10, −24	59	953	3.38
Inferior temporal gyrus	20	Left	−57, −57, −13	29	468	3.42

Whole-brain analysis involving one-sample *t*-tests with cluster thresholding with a *Z* threshold of 3.1 and *P* < 0.05. The areas indicated show greater activity in participants with cocaine use disorder than healthy control participants. MNI, Montreal Neurological Institute template.

**Fig. 3:**
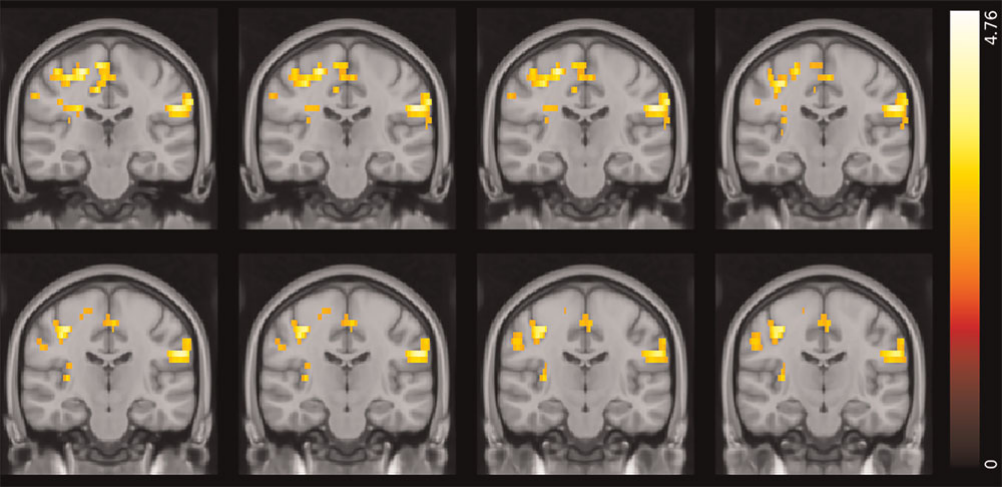
Punishment expected value tracking: differences between healthy controls and participants with gambling disorder (Montreal Neurological Institute coordinates: *Y* = −24 to −17). Activity was lower in the gambling disorder group in the areas indicated. Colour bar on the right-hand side represents the *t-*statistic.

For punishment expected value, we observed the opposite trend: individuals with gambling disorder showed lower activity in the superior parietal lobule, pre- and postcentral gyri, precuneus, parietal operculum, supramarginal gyrus and angular gyrus compared with healthy controls (Fig. 3 and Table 4). Activations were seen in both hemispheres, but were more pronounced in the right hemisphere.

**Table 4:** Summary of peak functional magnetic resonance imaging activity for the punishment expected value controls versus gambling disorder contrast

Name	Brodmann area	Side	MNI coordinates (*X, Y, Z*)	Number of voxels	Volume (mm^3^)	Mean *Z*-statistic
Postcentral gyrus	1, 2, 3	Right	56, −14, −33	1228	19 827	2.99
Postcentral gyrus	1, 2, 3	Left	−62, −21, −33	408	6588	3.02
Precentral gyrus	4	Right	43, −14, 45	865	13 966	2.95
Precuneus	7	Right	10, −51, 56	555	8961	2.90
Precuneus	7	Left	−6, −48, 56	403	6507	2.90
Superior parietal lobule	7	Right	28, −44, 59	524	8461	3.04
Supramarginal gyrus	40	Right	56, −18, 31	254	4101	2.88
Supramarginal gyrus	40	Left	−63, −26, 31	258	4166	3.02
Lateral occipital cortex	19	Right	17, −79, 41	242	3907	2.88
Lateral occipital cortex	19	Left	−14, −83, 41	181	2922	2.75
Parietal operculum cortex	40, 43	Right	1.5, −33, 21	106	1711	2.83
Parietal operculum cortex	40, 43	Left	−51, −33, 21	191	3084	2.98

Whole-brain analysis involving one-sample *t*-tests with cluster thresholding with a *Z* threshold of 3.1 and *P* < 0.05. The areas indicated show lower activity in participants with cocaine use disorder than healthy control participants. MNI, Montreal Neurological Institute template.

### Neural signal to positive and negative punishment prediction errors is altered in CUD

**Fig. 4:**
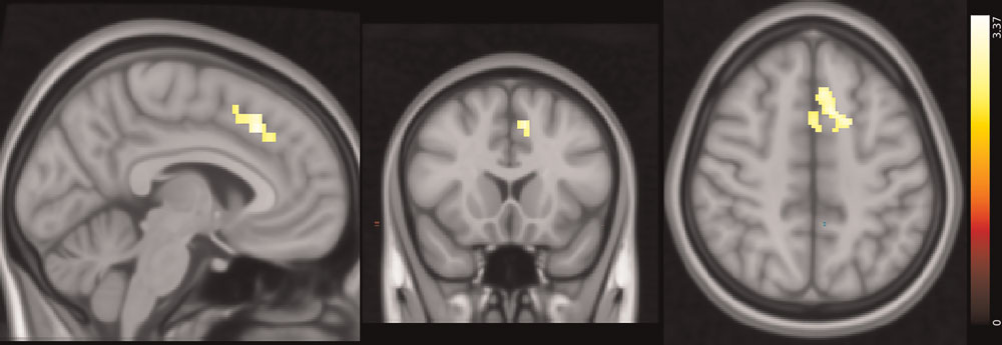
Response to positive punishment prediction errors: differences between healthy controls and participants with CUD (Montreal Neurological Institute coordinates: *X* = −5, *Y* = 17, *Z* = 48). Activity was lower in the CUD group in the areas indicated. Colour bar on the right-hand side represents the *t-*statistic. CUD, cocaine use disorder.

**Fig. 5:**
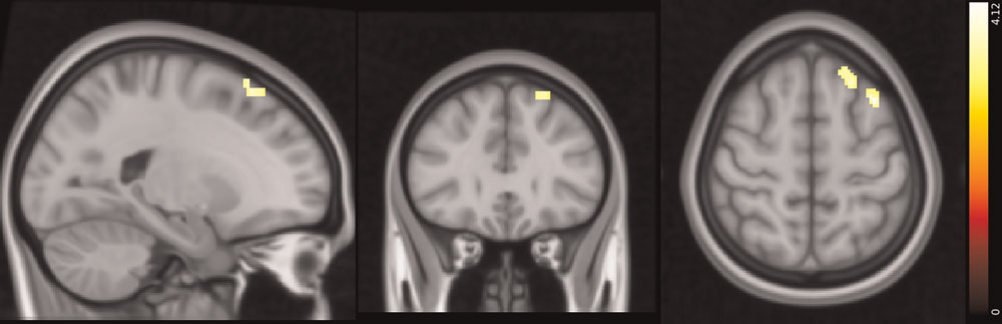
Response to negative punishment prediction errors: differences between healthy controls and participants with CUD (Montreal Neurological Institute coordinates: *X* = −31, *Y* = 30, *Z* = 56). Activity was higher in the CUD group in the areas indicated. Colour bar on the right-hand side represents the *t-*statistic. CUD, cocaine use disorder;

We observed aberrant neural responses in CUD as well, specifically in response to positive and negative PPEs. Compared with control participants, individuals with CUD exhibited lower activity in the paracingulate gyrus and left SFG in response to positive PPEs. Conversely, individuals with CUD showed greater activity in the left SFG and MFG in response to negative PPEs (Figs 4 and 5 and Tables 5 and 6, respectively).

**Table 5:** Summary of peak functional magnetic resonance imaging activity for the positive punishment prediction error controls versus cocaine use disorder contrast

Name	Brodmann area	Side	MNI coordinates (*X, Y, Z*)	Number of voxels	Volume (mm^3^)	Mean *Z*-statistic
Superior frontal gyrus	8, 9	Left	−10, 13, 53	149	2406	2.81
Paracingulate gyrus	32	Left	−8, 20, 43	132	2131	2.80
Paracingulate gyrus	32	Right	4, 11, 47	36	581	2.71

Whole-brain analysis involving one-sample *t*-tests with cluster thresholding with a *Z* threshold of 3.1 and *P* < 0.05. The areas indicated show lower activity in participants with cocaine use disorder than healthy control participants. MNI, Montreal Neurological Institute template.

**Table 6:** Summary of peak functional magnetic resonance imaging activity for the punishment prediction error controls versus cocaine use disorder contrast

Name	Brodmann area	Side	MNI coordinates (*X, Y, Z*)	Number of voxels	Volume (mm^3^)	Mean *Z*-statistic
Superior frontal gyrus	8, 9	Left	−57, −6, −17	71	1146	3.41
Middle frontal gyrus	8, 9	Left	−3, −66, 33	70	1130	3.41

Whole-brain analysis involving one-sample *t*-tests with cluster thresholding with a *Z* threshold of 3.1 and *P* < 0.05. The areas indicated show greater activity in participants with cocaine use disorder than control participants. MNI, Montreal Neurological Institute template.

Further results on the neural responses to feedback presentation can be found in the Supplementary Material.

### Whole-brain correlation analyses

The five parameters from the winning reinforcement learning model were used in a whole-brain correlation analysis to identify whether they correlated with the BOLD signal during each event type in any of the brain regions. This was done to identify the brain regions underlying reinforcement learning parameters. The first analysis related the parameters to activity from all participants.

This analysis highlighted that the *α*_rew_ parameter correlated negatively with activity in the cingulate and paracingulate gyri, inferior frontal gyrus (IFG), middle and superior temporal gyri, insular cortex and mOFC during reward expected value tracking, as well as responses to positive PPEs. This parameter also correlated negatively with activity in the putamen, mOFC and insula during positive RPEs.

Next, an ANCOVA was run to compare task-based activity among the different groups. In the gambling disorder group, *α*_rew_ correlated more strongly with activity in the SFG, MFG and postcentral gyrus during reward expected value tracking compared with the healthy control and CUD groups (Supplementary Fig. 3). In the CUD group, the correlation between *α*_rew_ and activity during the positive PPE was greater in the frontal pole, SFG, and cingulate and paracingulate gyri compared with the healthy control and gambling disorder groups (Supplementary Fig. 4).

**Fig. 6:**
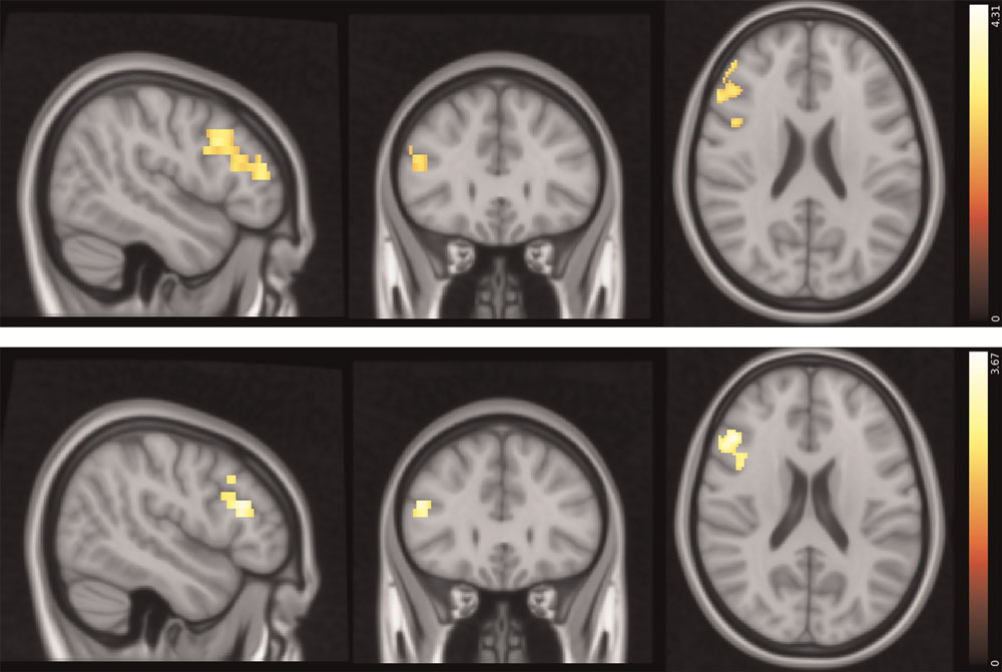
Top: areas that have a stronger positive correlation with *κ*_stim_ in the gambling disorder group than in the healthy control group (MNI coordinates: *X* = 48, *Y* = 29, *Z* = 22). Bottom: areas that have a stronger positive correlation with *κ*_stim_ in the CUD group than in the healthy control group (MNI coordinates: *X* = 48, *Y* = 29, *Z* = 20). Colour bar on the right-hand side represents the *t-*statistic. CUD, cocaine use disorder; MNI, Montreal Neurological Institute.

In both patient groups, stimulus stickiness (κ_stim_) had a stronger positive correlation with activity in the right MFG and IFG during response/feedback presentation compared with control participants, suggesting that there is greater activity in these areas in patients when repeating a response regardless of previous outcomes (Fig. 6). No other correlations with reinforcement learning parameters were found.

## Discussion

In this study, we examined reinforcement learning processes during a classic test of behavioural flexibility (PRL) in individuals with gambling disorder and CUD. Our computational modelling approach enabled the assessment of how both value-based (learning rates, reinforcement sensitivity) and value-free (stimulus and side stickiness) contributed to choice behaviour. The key behavioural result was that individuals with gambling disorder showed reduced choice repetition (stimulus stickiness), irrespective of the feedback received, suggestive of a maladaptive exploratory pattern. Reduced stimulus stickiness in gambling disorder contrasts with our recent observation of greater choice repetition in SUD, regardless of reinforcement.^13^ Our findings also extend the results presented in Verdejo-Garcia et al,^22^ which found a higher perseveration error rate in CUD and no differences in gambling disorder. We thus demonstrate that the use of reinforcement learning modelling can provide a novel insight into PRL data, and may help to explain which parameters contribute to differences in conventional measures. Stimulus stickiness (a form of choice repetition) may therefore present a novel way of dissociating compulsive disorders, in this case gambling disorder and CUD. However, we note that group differences were only observed at 75% HDI, but not at 95%. Furthermore, the sample sizes were relatively small.

We provide a novel and unexpected insight into how reinforcement learning parameters are affected in gambling disorder – that stimulus stickiness was reduced in this group. A similar reduction in stimulus stickiness has also been observed in another compulsive disorder, obsessive–compulsive disorder (OCD).^13^ However, in gambling disorder, the reduction in stimulus stickiness was accompanied by slightly higher levels of side stickiness *κ*_side_ (below 75% HDI), whereas in OCD there was additionally a mild reduction in side stickiness.^13^ In other words, the computational profile of gambling disorder and OCD appears to be distinct. Perseveration is not a unitary construct: side stickiness may be representative of motor perseveration, whereas stimulus stickiness reflects stimulus perseveration. Side stickiness may therefore represent excessive motor perseveration. In contrast, lower stimulus stickiness may reflect another form of behavioural inflexibility that is overly exploratory yet outcome insensitive. Low stimulus stickiness in gambling disorder detected during trial-and-error learning in a laboratory setting may therefore reflect a real-life increase in exploration of choices in an attempt to identify an optimal strategy, e.g. tracking new stimuli in a casino game. Overall, value-free contributors to choice behaviour have allowed for novel dissociations of gambling disorder, OCD and SUD, and point to a possible computational fingerprinting, which could eventually be useful for informing psychiatric classification.

At the neural level, group differences were also observed during ongoing reinforcement learning processes. Differences in brain activity when tracking reward and punishment expected values were seen in participants with gambling disorder. In these individuals, there was greater activity in response to reward expected values in areas including the amygdala, hippocampus and cingulate gyrus compared with healthy controls. When tracking punishment expected values, on the other hand, there was lower activity in regions such as the postcentral gyrus, superior parietal lobule and occipital areas, suggesting that individuals with gambling disorder differentially track expected values of stimuli in their surroundings in favour of reward-related expectancies. In the CUD group, there also appeared to be an altered balance in reinforcement learning, instead with lower responses to positive PPEs and greater responses to negative PPEs in the SFG and neighbouring regions compared with control participants, which suggests preferential processing of punishment. This aligns with our recent finding that individuals with SUD show greater punishment learning rates.^13^ We highlight the application of Bayesian statistics to the reinforcement learning modelling data compared with analyses of variance for the imaging data as a limitation of our analysis. In summary, there appear to be uniquely aberrant neural signals in each patient group when tracking value-related information important for reinforcement learning processes.

By linking the computational modelling parameters to the fMRI data, we also identified regions involved in the modulation of reinforcement learning measures, which has not been investigated in previous human studies. We found that the learning rate parameter for reward (*α*_rew_) was correlated with areas that responded to RPEs and PPEs, including the SFG, MFG, and cingulate and paracingulate gyri. Therefore, these regions appear to be of key importance for reinforcement learning and are likely to be involved in the modulation of the reward learning rate (*α*_rew_). The SFG and anterior cingulate cortex are key areas that have been shown to be involved in error and action monitoring, providing support for their involvement in reward learning.^29^ Moreover, a meta-analysis including 35 studies reported that these areas are consistently activated when there is a prediction error.^30^

At least two previous studies have reported reduced learning rates, reinforcement sensitivity and greater stimulus stickiness in individuals with SUD compared with healthy controls.^13,31^ In the present study, we observed lower reward learning rates and higher stimulus stickiness in CUD only when contrasted with gambling disorder. Duration of substance misuse may be a key factor underlying the less pronounced reinforcement learning results in CUD when compared with these two previous studies. Although the CUD sample in the present study had an average duration of substance use of 3.7 years,^22^ the participants with SUD in previous studies reporting more pronounced reinforcement learning deficits had an average duration of substance use of 11.7 years^6^ and 13.7 years.^31^ Additionally, a criterion in our study was abstinence, which was not the case in the other two investigations. These differences in sample suggest longer exposure to substances may have more pronounced effects on reinforcement learning processes, possibly because of neurotoxicity, and may therefore help reconcile the reinforcement learning findings between these studies. As gambling disorder itself does not involve substance use, we would not expect the same magnitude or mechanism of change in reinforcement learning effects related to disease duration (which was 2.2 years for gambling disorder in our sample). At the same time, such contrasts between gambling disorder and SUD may inform which aspects of reinforcement learning in SUD are more or less likely to be tied to neurotoxic effects.

Based on the neural results presented here, individuals with gambling disorder appear to be less sensitive to punishment expected value but more sensitive to reward expected value than controls. A study of performance on a two-choice lottery task found that choice behaviour in patients with gambling disorder was less sensitive to expected values for both reward and punishment, with this group using information about magnitude and probability information less than healthy controls.^32^ Thus, attenuated responses to punishment appear to be common across tasks in gambling disorder. Although greater sensitivity to reward was observed in our study compared with lower levels reported by Limbrick-Oldfield et al,^32^ this may have been because different behavioural paradigms were used. Consistent with our findings, in a previous study employing a card-guessing task, participants with gambling disorder had greater neural responses in the ventral striatum and OFC when tracking reward expected value.^33^ Overall, these studies suggest that patients with gambling disorder show altered responses to reinforcement tracking and are less sensitive to punishment.

In individuals with SUD, reduced responses to prediction errors in the ventral striatum and mOFC on the Iowa Gambling Task have been reported previously.^34^ In a separate study using electroencephalography, impaired RPE signalling in CUD was also found.^35^ In contrast, we found greater responses to PPEs, rather than reduction in RPEs. Following cocaine abstinence in individuals with CUD, enhanced signals to positive prediction errors, regardless of whether reward or punishment was predicted, have been observed.^36^ Although we report reduced activity following positive PPEs, this may be because we separated reward and punishment prediction errors, and suggests that the two prediction errors are differentially altered in CUD. Altered responses to prediction error related to both reward and punishment could be a contributor to compulsive drug use, as it persists despite negative outcomes. In patients with OCD, RPE responses were altered in the nucleus accumbens and anterior cingulate cortex, further highlighting that reinforcement learning can be used to distinguish disorders of compulsivity, both through behaviour and its associated neural substrates.^25^

We report that stimulus stickiness (*κ*_stim_) was positively correlated with activity in the dorsolateral PFC and vlPFC, areas important for cognitive control, including conflict monitoring and motor inhibition, respectively.^37^ In the results presented here, patients with gambling disorder and CUD showed a stronger positive correlation with stimulus stickiness (*κ*_stim_) in these regions. This result was contrary to our expectations and previous studies, as it was predicted that stickiness would be related to reduced activity in these regions. A possible interpretation of this finding is that stimulus stickiness reflects bias toward one of the presented stimuli, ideally the majority reinforced one, and that the MFG and IFG are active in order to overcome this response following a reversal. However, this hypothesis would need to be explored further in future studies.

It has been demonstrated previously that both the dorsolateral PFC and vlPFC are affected in gambling disorder and CUD;^38,39^ here, we provide a novel computational mechanism pertinent to compulsions that is linked to these regions in gambling disorder and CUD. Previous studies have demonstrated that response shifting on the PRL task is associated with vlPFC activation in control participants.^15^ Consistent with the present results, a prior analysis of this data-set showed the vlPFC was engaged during response shifting, yet both clinical groups showed lower vlPFC activity than healthy controls.^22^ Reduced vlPFC activity during shifting has been also reported in patients with OCD.^40^ These findings from previous studies, however, focus on response shifting on certain trials, whereas our analysis investigated stickiness across all trials, reflecting an overall tendency. Additionally, stickiness represents repeated responses, rather than response shifts. In rats, it has been shown that side stickiness (stimulus stickiness was not studied) is correlated with activity in medial PFC and dorsal striatal regions.^20^ It is therefore possible that side and stimulus stickiness recruit different neural circuits, but this requires further analysis in the same species.

In summary, we provide novel behavioural and neural insights into gambling disorder through computational modelling of reinforcement learning processes. Critically, we demonstrate that individuals with gambling disorder and CUD display perseverative behaviour during PRL that differs both qualitatively and quantitatively, advancing the notion that compulsivity is not a unitary construct. We also provide evidence that individuals with gambling disorder and CUD display aberrant and opposing neural responses to rewards and punishments, in relation to expected value and PPEs. Furthermore, we link reinforcement learning parameters to regions that may be involved in their modulation, which has not previously been investigated in the human literature. We demonstrate that reinforcement learning modelling combined with fMRI may provide new insights into the mechanisms underlying compulsive disorders, and therefore transdiagnostically refine our understanding of compulsivity.

## Supporting information

Zühlsdorff et al. supplementary material Zühlsdorff et al. supplementary material

## Data Availability

The data cannot be made publicly available due to the consent forms and ethics approval not explicitly stating that the data can be shared.
