# Supplementary material for: Computational modelling of reinforcement learning and functional neuroimaging of probabilistic reversal for dissociating compulsive behaviours in gambling and cocaine use disorders
Source: BJPsych Open. 2023 Dec 11;10(1):e8. doi: 10.1192/bjo.2023.611 (PMC10755559; doi:10.1192/bjo.2023.611)
Supplement: Zühlsdorff et al. supplementary material [file S2056472423006117sup001.docx]

Supplementary Methods

**Participant Recruitment**

Individuals with CUD were recruited in the outpatient clinic Centro Provincial de Drogodependencias, Granada, Spain, and participants diagnosed with GD were recruited from Asociación Granadina de Jugadores en Rehabilitación, Granada, Spain. Participants from these two groups had met the following inclusion criteria: 1) between 18-45 years old; 2) estimated IQ level above 80; 3) meeting the DSM-IV-TR criteria for cocaine dependence or pathological gambling; 4) having commenced psychological treatment; 5) having been abstinent for more than 15 days. It was confirmed that individuals with CUD were abstinent for three weeks during the study using two urine tests for alcohol and drug use per week and an additional test on the imaging day. Nicotine use was not controlled for, as participants were not tested for nicotine and abstinence was not required prior to the scan. Gambling abstinence was confirmed by relatives and checked through self-assessment. The following exclusion criteria were applied: 1) diagnosis of another Axis I or II disorder, except alcohol or nicotine addiction; 2) history of head injury, neurological disease or any other diseases affecting the central nervous system; 3) having undertaken other treatments in the two years prior to the study; 4) court-mandated treatment. The Structured Clinical Interview for DSM-IV Axis I Disorders (SCID-I-CV) was used to assess Axis I disorders, whereas Axis II disorders were assessed through the International Personality Disorders Examination (IPDE) (1,2). Diagnoses were made through registered clinical psychologists. Control participants were recruited from local agencies. Monthly amount and duration of drug use were also assessed using the Interview for Research on Addictive Behaviors (3). Participants had little exposure to drug other than cocaine, alcohol, and tobacco. A table with more details on this can be found in (4).

**Reinforcement Learning modeling**

The highest hierarchical level contained a group-specific mean and a common standard deviation for every RL parameter of the respective model. Priors for these values are shown in **Supplementary Table S1**. The RL parameters were drawn for each subject from a normal distribution having the relevant mean/standard deviation. Predicted choices were fit to behavior according to an RL algorithm (described below), and the highest posterior density interval (HDI) was calculated for group mean differences of interest (5).

The six models tested were as follows:

1. **Two parameters:** **α and β**, the learning rate and reinforcement sensitivity parameter.

2. **Three parameters: α,** **β,** stimulus stickiness parameter **κ_stim_**. κ_stim_ is the tendency to respond to the same stimulus as on the previous trial, irrespective of its location and outcome (i.e., whether it was rewarded or not), and was used to update the Q value as follows: ${Q^{stim}}_{s,t+1}=\kappa_{stim}S_{s,t}$. $S_{s,t}$ represents the stimulus chosen by the subject on the last trial. This value is 1 if the same stimulus was chosen as on the previous trial, and 0 if another stimulus was chosen. The final Q value is the sum of ${Q^{stim}}_{s,t+1}$ and the Q value as calculated in equation 1 (see main text).

3. **Three parameters: α_rew_, α_non-rew_, β.** Similar to model 1, but containing two separate learning rates for rewarded and non-rewarded trials, respectively.

4. **Four parameters: α_rew_, α_non-rew_, β and κ_stim_.**

5. **Four parameters: α_rew_, α_non-rew_, β and κ_side_.** The stimulus stickiness parameter was replaced with the side stickiness parameter, representing the tendency to choose the same side as on the previous trial, irrespective of the outcome produced, and was used to update the Q value as follows: ${Q^{loc}}_{s,t+1}=\kappa_{side}L_{s,t}$. $L_{s,t}$ represents the side chosen by the subject on the last trial. This value is 1 if the same side was chosen, and 0 if the other side was chosen. The final Q value is the sum of ${Q^{loc}}_{s,t+1}$ and the Q value as calculated in equation 1.

6. **Five parameters: α_rew_, α_non-rew_, β, κ_stim_ and κ_side_.**

The models were fitted through Hamiltonian Markov Chain Monte Carlo sampling via Stan 2.17.2 (6). Convergence was ensured using the potential scale reduction factor (7,8). A potential scale reduction factor value close to 1 indicated perfect convergence. A cut-off of 1.1 was selected as a stringent criterion for convergence. Models were compared and the winning model identified using a bridge sampling estimate of the marginal likelihood using the “bridgesampling” R package (9,10). The model with the highest marginal likelihood and posterior probability was considered to provide the best fit to the data.

Between-group differences were sampled to give a posterior probability distribution for each quantity of interest. These posterior distributions were interpreted using the 95% and 75% HDI, which are ‘credible intervals’ in Bayesian statistics. At 95% HDI, more evidence is provided for there being group differences than at 75% HDI. However, findings at 75% HDI are also considered to provide sufficient evidence for there being group differences.

**Data simulation**

Data were simulated using the posterior group mean parameters from the winning model, with the aim of determining whether the winning model could reproduce the behavioral observations. The simulated data were then analyzed using a conventional PRL analysis as described in (4). One hundred virtual “subjects” were simulated for each group, with each “subject” performing the PRL task in silico.

**Correlation between conventional and reinforcement learning measures**

Spearman’s rank coefficient was used to correlate conventional and RL parameters, as the data were non-normally distributed. Correction for multiple comparisons was accounted for using FDR correction (11). The statistical analyses were run in R version 4.0.4 (12). Significance was considered below p=0.05.

**Imaging acquisition**

Subjects were scanned in a 3T MRI scanner with an eight-channel phased-array head coil (Intera Achieva, Philips Medical Systems, Eindhoven, The Netherlands). First, three T2*-weighted scans using an echo planar imaging (EPI) sequence were taken (repetition time (TR)=2000 ms, time to echo (TE)=35 ms, field of view (FOV)=230x230 mm, 96x96 matrix, flip angle=90°, 21 4-mm axial slices, 1-mm gap, 330 scans each). Subsequently, a sagittal three-dimensional T1-weighted turbo-gradient-echo sequence was used (150 slices, TR=8.3 ms, TE=3.8 ms, flip angle=8°, FOV=240x240, 1 mm^3^ voxels). More details can be found in (4).

**Image pre-processing**

The FMRIB Software Library (FSL) and FMRIPREP were used to pre-process the data (13,14). FMRIPREP implements multiple software, including FSL and the Advanced Normalisation Tools (ANTs) (15). Each T1-weighted image was bias-field corrected using *N4BiasFieldCorrection* and skull-stripped using *antsBrainExtraction* with the OASIS template from the ANTs software. Functional MRI scans were spatially normalized to the ICBM 152 Nonlinear Asymmetrical template version 2009c through non-linear registration with the *antsRegistration* tool using brain-extracted versions of both the T1-weighted (T1w) volume and template (16).

Subsequently, brain extracted T1w images were segmented into cerebrospinal fluid, white matter and grey matter using *fast* (FSL) (17). Functional MRI scans were slice-timing-corrected using *slicetimer* (FSL) and then motion-corrected with *mcflirt* (FSL) (18). For scans with associated field maps, distortion correction was performed using *fugue* (FSL) (19). Next, the fMRI images were co-registered to their corresponding T1w scan using boundary-based registrations with six degrees of freedom with *flirt* (FSL) (20). The field distortion correcting warp, BOLD-to-T1w transformation and T1w-to-template (MNI) warp were concatenated and applied in a single step using *antsApplyTransforms* using Lanczos interpolation. Nipype was used to calculate the frame-wise displacement (21). The first five volumes were discarded to avoid T1 saturation effects. fMRI images were high-pass filtered (128 s) and spatially smoothed with a 6 mm full-width, half-maximum 3D Gaussian kernel. A canonical hemodynamic response function was modelled to the onsets of the explanatory event types. Multiple criteria were used to ensure successful registration, including checking successful registration, ensuring that none of the participants showed excessive motion using DVARS (root mean square of the temporal change of the voxel-wise signal at each time point (22)) and framewise-displacement measures (excessive motion threshold being 10% of the total number of volumes) and by inspecting their respective carpet plots. No participants were found to have excessive motion.

Supplementary Results

**Relationship between conventional and reinforcement learning parameters**

The learning rate from rewarded trials $\alpha_{rew}$ was positively correlated with win-stay and lose-shift as well as the proportion of correct responses (p<0.001, p<0.001 and p=0.007, respectively). This parameter was also negatively correlated with the number of trials required to reach criterion (p<0.001). $\alpha_{non-rew}$, the learning rate from non-rewarded trials correlated negatively with the proportion of correct responses (p=0.007) and win-stay behavior (p=0.048), correlated positively with trials to criterion (p=0.005) and lose-shift behavior (p<0.001, respectively). The exploitation vs exploration parameter $\beta$ was correlated with all parameters except lose-shift behavior and the number of perseverative responses. It was positively correlated with the proportion of correct responses (p<0.001) and win-stay behavior (p<0.001), and negatively correlated with trials to criterion (p<0.001). $\kappa_{side}$ was not correlated with any of the measures. $\kappa_{stim}$, on the other hand, was negatively correlated with lose-shift behavior (p<0.001). For more details, see (23).

**Neural responses to feedback presentation**

During feedback presentation, the GD group overall showed increased activity (versus controls) in the lateral occipital cortex, cingulate gyrus, parahippocampal gyrus, precuneus, middle temporal gyrus and supramarginal gyrus (supplementary materials, **Figure S1**). There were also significantly greater activations during feedback presentation in the CUD group (versus controls), which were instead in the frontal pole, SFG, inferior frontal gyrus (IFG), precentral gyrus, superior parietal lobule, supramarginal gyrus, precuneus, angular gyrus, and lateral occipital cortex (**Figure S2**). Moreover, we observed differences between the CUD and GD groups: individuals with CUD had greater activity than those with GD in the insular cortex, IFG, and frontal operculum.

No significant differences were found in response to positive and negative RPEs. Thus, there appear to be widespread differences in both CUD and GD groups when the feedback was presented. However, this response was altered in different areas of the brain in the two disorder groups.

**Supplementary Table S1.** Priors for model parameters.

| **Parameter** | **Prior** | **Reference** |
| --- | --- | --- |
| Reward learning rate, $\alpha_{rew}$ | Beta(1.2, 1.2) | (24) |
| Punishment learning rate, $\alpha_{pun}$ | Beta(1.2, 1.2) | (24) |
| Combined learning rate, $\alpha$ | Beta(1.2, 1.2) | (24) |
| Reinforcement sensitivity, β | Gamma(α=4.82, β=0.88) | (25) |
| Side stickiness, κ_side_ | Normal(0,1) | (26) |
| Stimulus stickiness, κ_stim_ | Normal(0,1) | (26) |
| **Intersubject variability in parameters** |  |  |
| $\alpha_{rew}$, $\alpha_{pun}$, $\alpha$, κ_side,_ κ_stim_ intersubject standard deviations | Normal(0,0.05) constrained to $\geq$0 | (27) |
| β intersubject standard deviations | Normal(0,1) constrained to $\geq$0 | (25) |

**Figure S1.** Feedback/cue presentation: differences between healthy controls and participants with GD (MNI coordinates: X=31, Y=-68, Z=27). Activity was higher in the GD group in the areas indicated. Color bar on the right-hand side represents *t*.

**
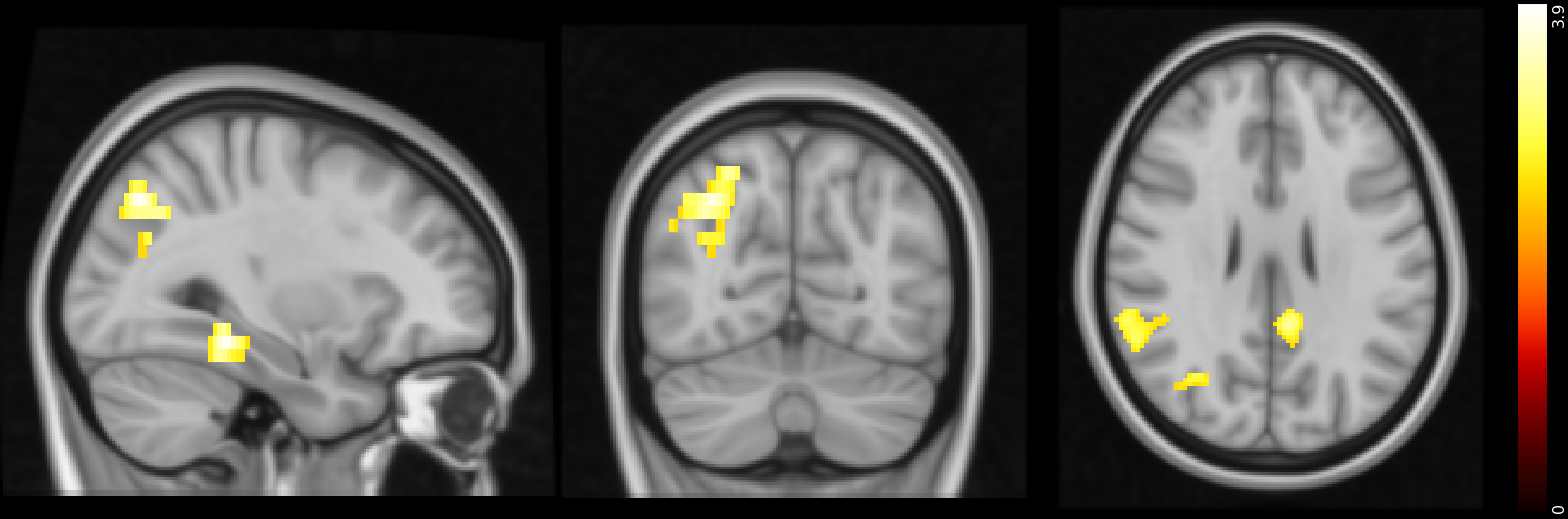
**

**
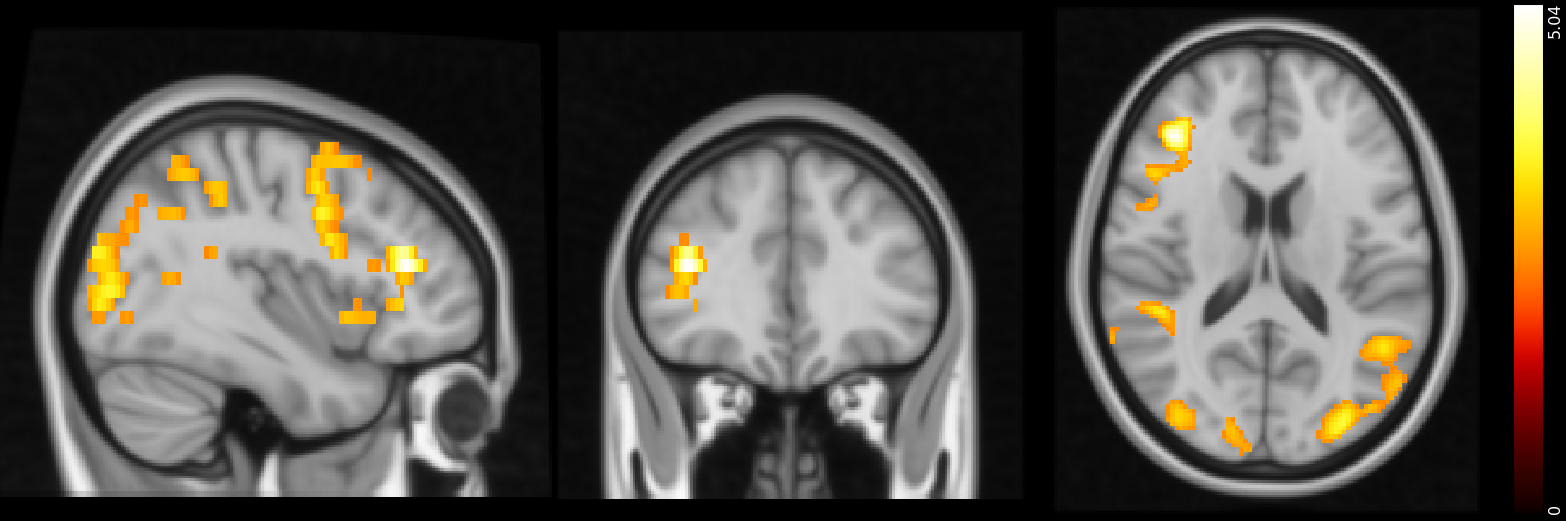
Figure S2.** Feedback/cue presentation: differences between healthy controls and participants with GD (MNI coordinates: X=39, Y=34, Z=17). Activity was higher in the CUD group in the areas indicated. Color bar on the right-hand side represents *t*.

**Figure S3.** Areas that have a stronger negative correlation with α_rew_ in the GD group than in

healthy controls during reward EV tracking (MNI coordinates: X=-32, Y=12, Z=52). Color


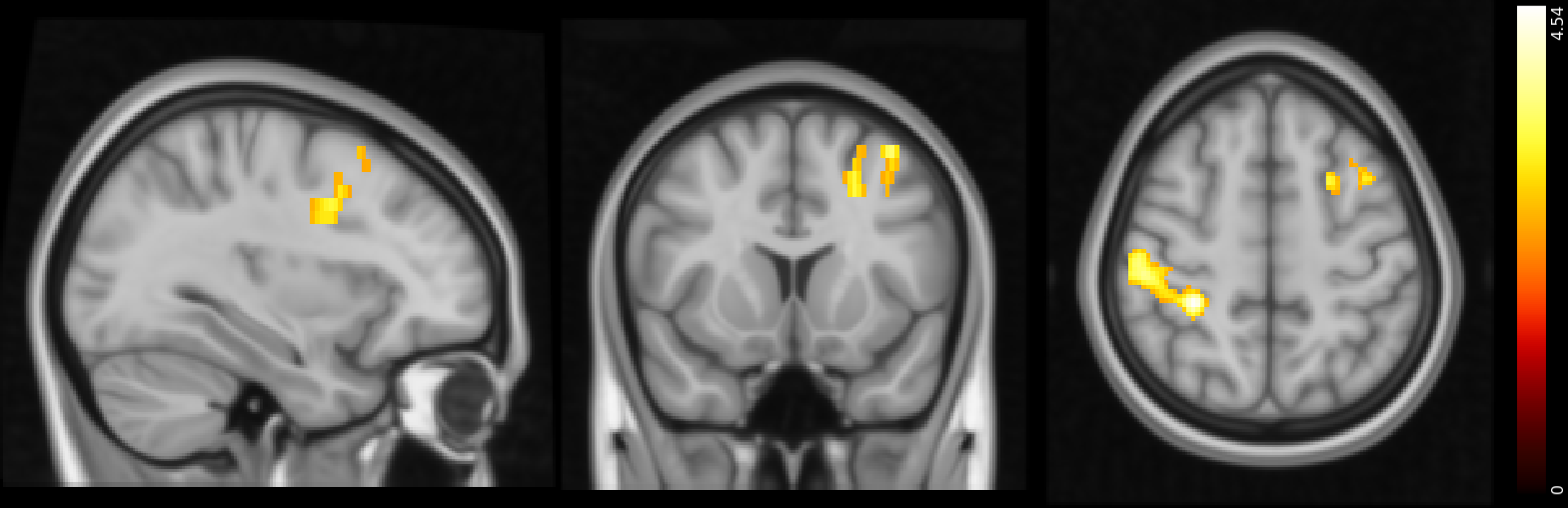
bar on the right-hand side represents *t*.

**Figure S4.** Areas that have a stronger negative correlation with α_rew_ in the CUD group than in

**
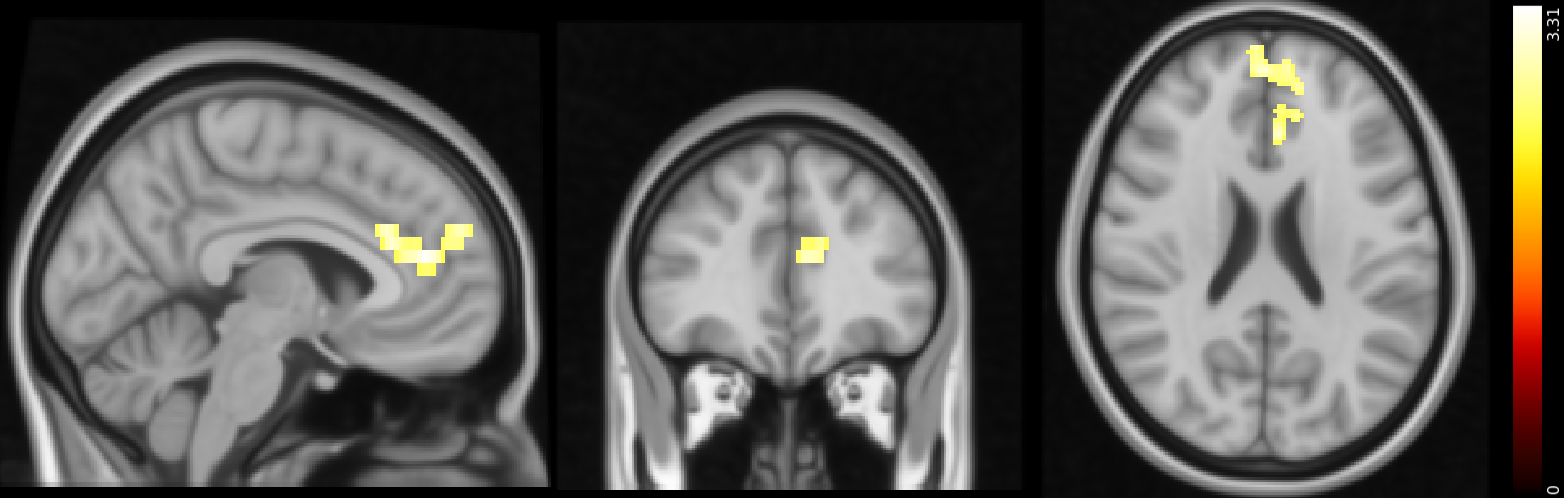
**healthy controls when responding to positive PPE (MNI coordinates: X=-5, Y=37, Z=22). Color bar on the right-hand side represents *t*.

**Figure S5.** Simulations of behavior on the probabilistic reversal learning task based on reinforcement learning parameters. No group differences were found on any of the parameters, including the proportion of correct responses, trials to criterion and perseverative responses. For a direct comparison to the original behavioral data, see (4).


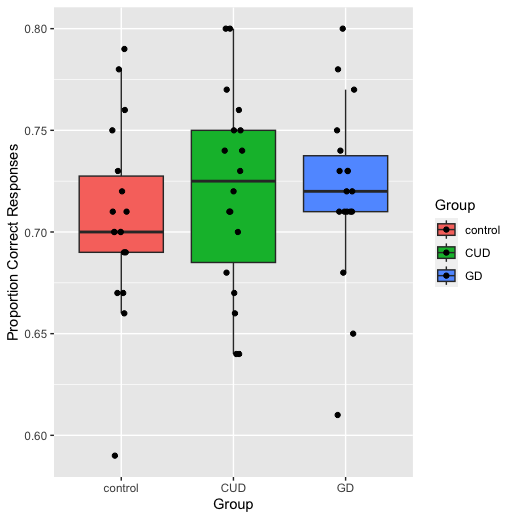

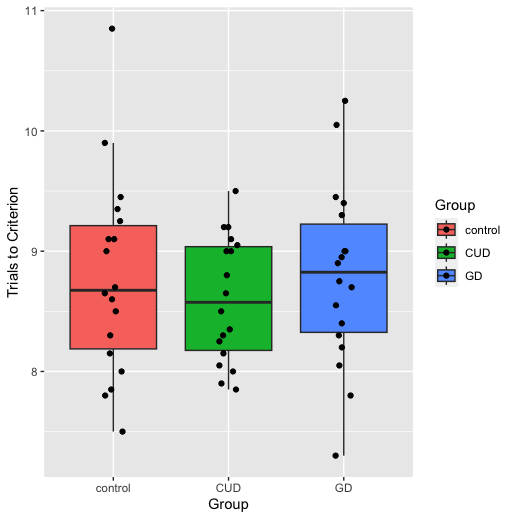

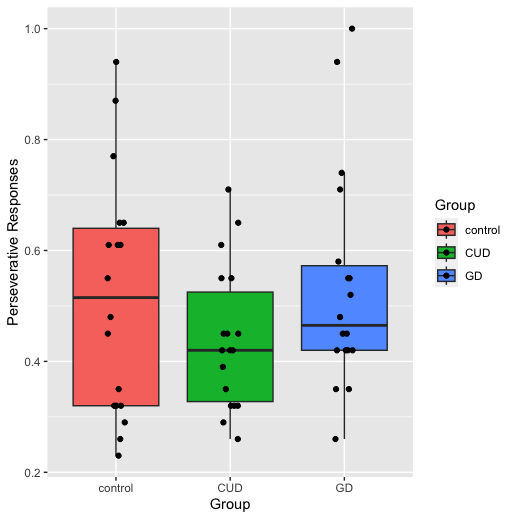


References

1. First MB. Structured clinical interview for DSM-IV axis I disorders. Biometrics Res Dep. 1997;

2. Loranger AW. The International Personality Disorder Examination. Arch Gen Psychiatry. 1994;51(3).

3. López-Torrecillas F, García JFG, García MP, Izquierdo DG, Sánchez-Barrera MB. Variables modulating stress and coping that discriminate drug consumers from low or nondrug consumers. Addict Behav. 2000 Jan;25(1):161–5.

4. Verdejo-Garcia A, Clark L, Verdejo-Román J, Albein-Urios N, Martinez-Gonzalez JM, Gutierrez B, et al. Neural substrates of cognitive flexibility in cocaine and gambling addictions. Br J Psychiatry [Internet]. 2015 Aug 1 [cited 2020 Dec 8];207(2):158–64. Available from: https://pubmed.ncbi.nlm.nih.gov/26045346/

5. Kruschke JK. Doing Bayesian data analysis: A tutorial with R, JAGS, and Stan, second edition. Doing Bayesian Data Analysis: A Tutorial with R, JAGS, and Stan, Second Edition. 2014.

6. Carpenter B, Gelman A, Hoffman MD, Lee D, Goodrich B, Betancourt M, et al. Stan: A Probabilistic Programming Language. J Stat Softw [Internet]. 2017 Jan 11 [cited 2022 May 27];76(1):1–32. Available from: https://www.jstatsoft.org/index.php/jss/article/view/v076i01

7. Brooks SP, Gelman A. General methods for monitoring convergence of iterative simulations)? J Comput Graph Stat. 1998;7(4).

8. Gelman A. Bayesian data analysis. Vol. 53, Journal of Chemical Information and Modeling. 2013.

9. Gronau QF, Van Erp S, Heck DW, Cesario J, Jonas KJ, Wagenmakers EJ. A Bayesian model-averaged meta-analysis of the power pose effect with informed and default priors: the case of felt power. Compr Results Soc Psychol. 2017;2(1).

10. Gronau QF, Singmann H, Wagenmakers EJ. Bridgesampling: An R package for estimating normalizing constants. J Stat Softw. 2020;92.

11. Benjamini Y, Hochberg Y. Controlling the False Discovery Rate: A Practical and Powerful Approach to Multiple Testing. J R Stat Soc Ser B. 1995;57(1).

12. R Core Team. R: A Language and Environment for Statistical Computing. R Foundation for Statistical Computing. 2021.

13. Esteban O, Markiewicz CJ, Blair RW, Moodie CA, Isik AI, Erramuzpe A, et al. fMRIPrep: a robust preprocessing pipeline for functional MRI. Nat Methods 2018 161 [Internet]. 2018 Dec 10 [cited 2021 Jul 27];16(1):111–6. Available from: https://www.nature.com/articles/s41592-018-0235-4

14. Smith SM, Jenkinson M, Woolrich MW, Beckmann CF, Behrens TEJ, Johansen-Berg H, et al. Advances in functional and structural MR image analysis and implementation as FSL. In: NeuroImage. 2004.

15. Tustison NJ, Avants BB, Cook PA, Zheng Y, Egan A, Yushkevich PA, et al. N4ITK: Improved N3 bias correction. IEEE Trans Med Imaging. 2010 Jun;29(6):1310–20.

16. Avants BB, Epstein CL, Grossman M, Gee JC. Symmetric diffeomorphic image registration with cross-correlation: Evaluating automated labeling of elderly and neurodegenerative brain. Med Image Anal. 2008 Feb 1;12(1):26–41.

17. Zhang Y, Brady M, Smith S. Segmentation of brain MR images through a hidden Markov random field model and the expectation-maximization algorithm. IEEE Trans Med Imaging. 2001 Jan;20(1):45–57.

18. Jenkinson M, Bannister P, Brady M, Smith S. Improved Optimization for the Robust and Accurate Linear Registration and Motion Correction of Brain Images. Neuroimage. 2002 Oct 1;17(2):825–41.

19. Jenkinson M. Fast, automated, N-dimensional phase-unwrapping algorithm. Magn Reson Med [Internet]. 2003 Jan 1 [cited 2021 Jul 27];49(1):193–7. Available from: https://onlinelibrary.wiley.com/doi/full/10.1002/mrm.10354

20. Greve DN, Fischl B. Accurate and robust brain image alignment using boundary-based registration. Neuroimage. 2009 Oct 15;48(1):63–72.

21. Power JD, Mitra A, Laumann TO, Snyder AZ, Schlaggar BL, Petersen SE. Methods to detect, characterize, and remove motion artifact in resting state fMRI. Neuroimage. 2014 Jan 1;84:320–41.

22. Yang Z, Zhuang X, Sreenivasan K, Mishra V, Cordes D, Initiative the ADN. Robust Motion Regression of Resting-State Data Using a Convolutional Neural Network Model. Front Neurosci. 2019 Feb 28;13:169.

23. Zuhlsdorff K. Investigating reinforcement learning processes in depression and substance use disorder: translational, computational and neuroimaging approaches. 2022 Jul 19 [cited 2022 Dec 19]; Available from: https://www.repository.cam.ac.uk/handle/1810/343811

24. Den Ouden HEM, Daw ND, Fernandez G, Elshout JA, Rijpkema M, Hoogman M, et al. Dissociable effects of dopamine and serotonin on reversal learning. Neuron [Internet]. 2013 Nov 20 [cited 2022 Jul 14];80(4):1090–100. Available from: https://pubmed.ncbi.nlm.nih.gov/24267657/

25. Gershman SJ. Empirical priors for reinforcement learning models. J Math Psychol [Internet]. 2016 [cited 2022 Jul 14];71:1–6. Available from: http://dx.doi.org/10.1016/j.jmp.2016.01.006

26. Christakou A, Gershman SJ, Niv Y, Simmons A, Brammer M, Rubia K. Neural and psychological maturation of decision-making in adolescence and young adulthood. J Cogn Neurosci [Internet]. 2013 [cited 2022 Jul 14];25(11):1807–23. Available from: https://pubmed.ncbi.nlm.nih.gov/23859647/

27. Kanen JW, Ersche KD, Fineberg NA, Robbins TW, Cardinal RN. Computational modelling reveals contrasting effects on reinforcement learning and cognitive flexibility in stimulant use disorder and obsessive-compulsive disorder: remediating effects of dopaminergic D2/3 receptor agents. Psychopharmacology (Berl) [Internet]. 2019 Aug 1 [cited 2021 Mar 16];236(8):2337–58. Available from: https://doi.org/10.1007/s00213-019-05325-w
